# P-1409. Differential Impact of a Penicillin Allergy Assessment Tool on Orthopedic Surgical Patients from Areas of High Social Vulnerability

**DOI:** 10.1093/ofid/ofae631.1584

**Published:** 2025-01-29

**Authors:** Catherine Passaretti, Michael S Boger, Rupal K Jaffa, Anupama Neelakanta, Michael Inman, Lisa Davidson

**Affiliations:** Advocate Health, Charlotte, North Carolina; Atrium Health, Charlotte, North Carolina; Atrium Health, Charlotte, North Carolina; Atrium Health, Charlotte, North Carolina; Atrium Health, Charlotte, North Carolina; Atrium Health, Charlotte, North Carolina

## Abstract

**Background:**

Penicillin allergy labels (PAL) have been associated with increased risk of surgical site infections. Many penicillin allergy assessment tools are being trialed to improve perioperative β-lactam utilization. We report on the sociodemographic characteristics associated with receipt of β-lactam antibiotics (BLA) in orthopedic surgical patients with a PAL after implementation of a penicillin allergy assessment tool built into the electronic health record (EHR).

**Table 1:**
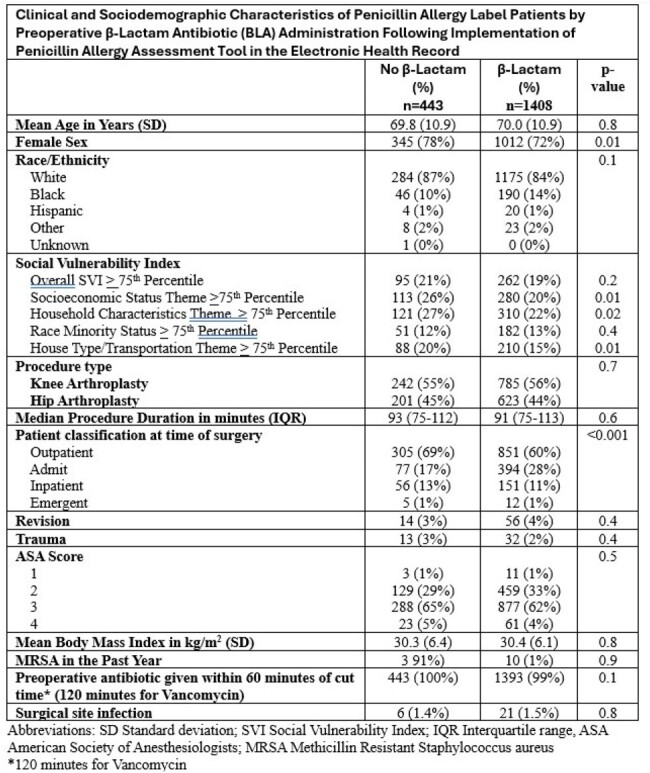
Clinical and Sociodemographic Characteristics of Penicillin Allergy Label Patients by Preoperative β-Lactam Antibiotic (BLA) Administration Following Implementation of Penicillin Allergy Assessment Tool in the Electronic Health Record

**Methods:**

We performed a retrospective cohort study of patients who underwent hip (HPRO) and knee arthroplasty (KPRO) procedures in a large health system between August 2022 and December 2023. Patients with a PAL were identified and demographic, clinical and antibiotic administration data were obtained from the EHR. Clinical data was merged with Social Vulnerability Index (SVI) census-track data comprising 4 subthemes (socioeconomic status, household characteristics, racial/ethnic minority status and housing type/transportation). Higher SVI indicates higher vulnerability. Patients with a PAL who did and did not receive a BLA post protocol implementation were compared.

**Figure 1:**
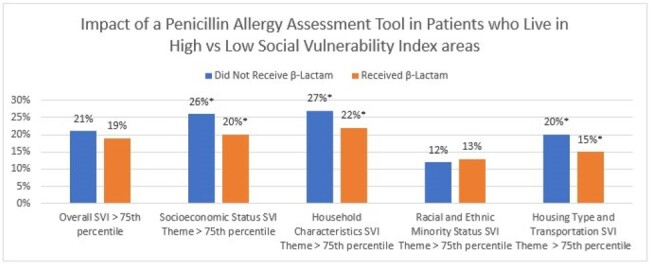
Impact of a Penicillin Allergy Assessment Tool on Beta Lactam Utilization in Patients who Live in High vs Low Social Vulnerability Index Census Tracks *Statistically significant p<0.05

**Results:**

1851 patients with a PAL who had undergone a HPRO and/or KPRO were identified. After implementation of the penicillin allergy assessment tool, 443 (24%) did not receive a BLA preoperatively. PAL Patients that did not receive a BLA after implementation of the tool were more likely to be female and less likely to have an admit status at the time of surgery. Other clinical characteristics including surgical site infection rates were similar between the groups. (Table 1) PAL patients who did not receive BLA were more likely to live in areas with higher socioeconomic status SVI (26% vs 20%), higher household characteristics SVI (27% > vs 22%), and higher housing type SVI (20% vs 15%) than those who did receive BLA. (Figure 1)

**Conclusion:**

Following implementation of an electronic penicillin assessment tool, most patients with a PAL were able to receive BLA safely. The subset of PAL patients who still did not receive BLA more frequently lived in areas with increased social vulnerability. Additional efforts may be needed to ensure the full benefit of tools is achieved in operative patients from areas with high SVI.

**Disclosures:**

**All Authors**: No reported disclosures

